# Extracts and Gleanings

**Published:** 1875-06

**Authors:** 


### Extracts and Gleanings.

---

*Jaborandi.*—(*Polycarpus Pinnatus.*)—This new and powerful remedy, with which all the journals are teeming, seems destined to come into very extensive use, and throws all our pet diaphoretics and sialagogues as much in the shade as the Whitworth rifle does the old tower musket. Only a very small supply has been yet obtained, and none, as yet, appears to have been brought to this country. The supply now in use was brought from Pemambuco; but there are other plants called jaborandi by the natives that do not partake of the active principles of the polycarpus pinnatus. The effects, as far as they have been observed, consist in profuse flow of saliva, very energetic diaphoresis, flushing of face and neck, increase of heart's action, dimness of vision, and vomiting. Pain over the pubes has been met with, but is immediately relieved by passing water, and appeared to be caused by a contraction of the bladder, as the amount of urine was not increased. When the leaves are chewed, the taste is piquant and excites a glowing heat on the tongue. It also acts as an external irritant. The temperature is always depressed from 0.4° to 1.5°, but the depression, Dr. Ringer says, does not last for more than a few hours. The antagonism between jaborandi and belladonna is very marked. The injection of atropia under the skin stops the flow of sweat produced by jaborandi, and in case of poisoning by atropia the symptoms all disappeared under its use. This drug has little or no effect on the bowels, and, like belladonna, children are far less subject to it than adults; in fact, Dr. Ringer's experiments on seventeen children gave almost negative results. Its action on the skin and salivary glands is almost remarkable. Dr. Martindale experimented on himself with sixty grains, made into an infusion and taken, with the dregs. In five minutes the action began, and continued for two hours. He says the Turkish bath was nothing to it, and he measured sixteen ounces of saliva, besides a large quantity that was lost. In one of Dr. Ringer's experiments it amounted to twenty-seven ounces.

Jaborandi also has a marked effect on the milk, increasing it from forty minims, in ten minutes, to one hundred and fifty-five; but the effect was not lasting, the secretion returning to its normal in about two hours.

Those who have experimented with it are as yet uncertain of its mode of action, some attributing it to partial paralysis of the vaso-mortor nerves, thus increasing the caliber of the smaller blood-vessels, and others assert that it is a direct stimulant to the secreting glands.

The indications for its employment cannot as yet be fully stated. Prof. Lubler (*Gazette Hebdom.*, February 19, 1875) says he has found serous effusions diminish, and has cut short attacks of influenza. He has found it very useful in chronic bronchitis, emphysema, with asthmatic paroxysms.

It would seem to be indicated in all cases of general anasarca and œdema from rheumatism, albuminuria, or diseases of the heart. Pleuritic effusions and most febrile states will be benefited by it.

The dose varies from thirty to sixty grains of the powdered leaves; the infusion, tincture and fluid extract are also active.

This is a very brief abstract from several very interesting papers on this drug, as our space is limited; but one of our enterprising druggists has sent for some of the article, and we hope soon to present to our readers a full history of it, with experiments.

H. B. L.

*Tincture of Iodine for Cloasma Uterina.*—Dr. Dubois recommends this method of treating the unsightly patches that so frequently disfigure the faces of pregnant women. Every evening a coating of the tincture is to be applied to the spots. The epidermis exfoliates and the spots disappear. If this does not follow the first application and some pain results, he then suspends the use of the iodine and replaces it with cold cream. Then, when the epidermis is newly formed, he recommences the use of the iodine, and this time the patch will disappear entirely.—*Gaz. Hebdom.*

*On the Treatment of Diarrhœa of Typhoid Fever.*—In a clinical lecture on this subject by Dr. Johnson, of King's College Hospital, he says :

" I have gradually arrived at the conclusion that in the treatment of typhoid fever careful nursing and feeding are of primary importance, while as a rule no medicines of any kind are requried, and when not required they are often worse than useless. The result of this change of treatment has been that diarrhœa is a less frequent symptom than formerly, and when it does occur it is far more tractable, while tympanitic distension of the abdomen is a rare event. The mischievous opiate enemata and the torturing turpentine stupes have disappeared together. I believe that one of the main reasons why we have less diarrhœa than formerly is that we carefully abstain from the employment of irritating drugs of all kinds. As a rule, a fever patient has the 'yellow mixture,' which is simply colored water; and except an occasional dose of chloral to procure sleep, and a tonic during convalescence, we give no active medicines of any kind. We feed these patients mainly with milk, with the addition of beef-tea and two raw eggs in twenty-four hours, and we give wine or brandy in quantities varying according to the urgency of the symptoms of exhaustion, especially in the advanced stages of the disease; but in many of the milder cases, and especially in the case of children, we find that no alcoholic stimulants are required from the beginning to the end of the fever, and when not required they are of course best withheld. I have said that we give no irritating drugs of any kind. For a time I adopted the practice, which has been strongly recommended, of giving repeated doses of diluted mineral acids. I have long since abandoned this practice, for I am sure that it was injurious, and it was injurious in a very obvious and intelligible way ; it irritated the ulcerated mucous membrane of the intestines; it caused pain and griping; and I believe that it often increased the diarrhœa. I have no doubt that the comparative infrequency of severe and obstinate diarrhœa among my enteric-fever patients during the last few years is partly attributable to the discontinuance of this mineral acid treatment.

" The diarrhœa of typhoid fever is in all probability often increased by the patient's inability to digest the beef-tea and eggs, which are sometimes too abundantly given. When you have reason to suspect that this may be the case, I advise you for a few days to keep the patient upon milk, which contains all the elements required for the nutrition of the tissues in a form most easy of digestion. Milk has an antilaxative and even constipating effect in various morbid states, and is, when given alone, one of the best antidotes for the diarrhœa of typhoid fever."—*American Practitioner.*

*Preventive Medicine.*—By Prof. R. C. KEDZIE, M. D.—Abstract of an Address to the Graduating Class, Medical Department of Michigan University, Ann Arbor, March 24, 1875.—Preventive medicine is now pressing its claims with an emphasis never before heard. The race demands of the profession not only to repair the ravages of disease, but to save them from its power. Follow the phalanx of human life as it marches on, and see how fast its ranks are depleted by deserters, till we arrive at three-score years, only to find a body-guard left. Admit that there are fixed climatic conditions unfavorable to long life; that accident and unavoidable conditions destroy a certain per cent.; pass over all the victims whom human foresight and prudence could not save, and what a fearful host still remains, cut off in their vigor and prime by preventable causes. See consumption, like the hovering wings of the angel of death, overshadowing the race; cholera and yellow fever sweeping over the land and sowing the earth thick with graves; intemperance sending body and soul to the demon's hell; typhus and typhoid gathering the sheaves of the harvest of death; consider these and a score beside, and tell me if there is no work to be done in the fields of preventive medicine. "Lift up your eyes and look on the fields, for they are white already to harvest." The old superstitions which connected unusual sickness with the wrath of offended Deity have faded in the light of science. The black death, cholera, typhus, scurvy, etc., are as truly the penalties of violated sanitary laws as is death by submersion in water.

As science sheds more fully its light upon these dark and perplexing questions, we see more and more clearly that sickness and pain are the fruits of our own misdoings. The "misterious providences" about which we have heard so much are resolving themselves into "defective drainage," "sewage contamination," "unwholesome food," "poisoned walls," "no ventilation," etc.

The school-rooms, the lecture-halls, the court-rooms, the temples of religion, the halls of legislation, the hospitals, the prisons, and even our sacred bed-chambers, are full of subtle poison.

Go into our cities and villages and see the festering graveyards pouring their literally deadly contents into every well in the vicinity; the cesspools pouring rectified death through all the subsoil; an epidemic of dysentery or typhoid fever sweeps over the afflicted community, and men bow themselves before "the mysterious providence" and roll up their eyes as though they were objects worthy of deepest pity. Away with the impiety which would flout our filth in the face of Deity and say that these afflictions come from His hand. The voice of God thunders as of old, "Wash you, and be ye clean," if you expect His favor—clean in your person and homes, the food you eat, the water you drink, and the air you breathe; clean in thought and in life.—*The Sanitary.*

*Pruritus of the Genitals.*—Dr. M. B. Wright stated that he had lately been called to see a lady suffering from pruritus of the genitalia. She was between two and three months advanced in pregnancy. Two years ago he had attended her when suffering with the same symptoms and under the same circumstances. She told him that she had borne four or five children, and that she had learned to recognize this intense itching as one of the first positive signs of pregnancy. At the time he attended her, two years ago, he had made various applications, but these had afforded but partial relief, and now her itching lasted throughout the entire period of utero-gestation. In that case the only means by which the woman could obtain any relief and rest was by introducing pounded ice into the vagina at night. During the day it became so much intensified upon the motions incident to her household labors as to compel her to withdraw from all society, even at times from her own family. In the case now under treatment he was using a 20 gr. solution of carbolic acid through the speculum, and the patient herself also applied it to the vulva. He asked for the experience or suggestions of members with reference to these troublesome cases.

Dr. Reamy said that the treatment which he had adopted for the last two years was founded on the belief that the irritation in these cases was caused by a discharge from the cervix, just as in the non-pregnant varieties. During this period he had observed pruritus in the cases of three pregnant women, in all of whom there was a slight discharge from the os, caused by an excitement of the cervical glands, an excitement resulting in some cases from pregnancy, and in all of whom the irritation had ceased after the stoppage of this discharge. A weak solution of nitrate of silver pushed up the cervical canal so as to come in contact with the glands would have the desired effect. In reply to a question by Dr. Wright, he said that in his opinion such a solution could be applied with perfect safety at least half way up the cervix. In these cases connected with pregnancy the pruritus does not generally depend upon trouble in the vulva, vagina, or vaginal portion of the cervix, but according to his belief, upon the very small amount of discharge from the cervical glands. In one case he had used the sulphate of zinc; it is immaterial what agent be employed, provided the excitement of these glands be checked.

Dr. Isham reported uniform success with the following formula: Carbolic acid, gr. x.; glycerine, ℥ss.; and brown citrine ointment, ℥ss.

Dr. Buckner reported a case in which, after various other remedies had proved failures, the local application of a strong solution of bichloride of mercury had entirely relieved the distressing symptoms.

Dr. C. O. Wright stated that he was now treating a case of excessive pruritus of six months standing, by means of the oil of spruce tar. The results thus far have been very gratifying.

Dr. M. B. Wright recalled the attention of the members to the fact that his case depended entirely upon pregnancy, as it had appeared at no other times. Was now treating a young married lady five months pregnant. Several days ago she complained of some swelling about the external genitals. Examination disclosed a varicose condition of all the veins of the surface, and particularly in the region of the meatus urinarius. He suspected that this condition might perhaps be the foundation of more of these cases.—*Clinic.*

*The Prevention of Sea-sickness.*—Dr. Giraldes has published, in the last number of the *Journal de Therapeutique*, an account of the means by which he avoided sea-sickness during two passages to

England and back. He was at Boulogne last June *en route* for London, when the weather was so rough that many intending passengers hesitated to cross the channel. Dr. Giraldes was informed by a colleague at Boulogne that American physicians used the syrup of chloral as a preventive of sea-sickness with successful results. He therefore obtained some syrup of chloral, put himself into a quiet corner, and took his syrup directly the vessel was in motion, when although his fellow passengers experienced the usual unpleasant consequences, he arived at Folkestone without having suffered the least inconvenience. The same results were obtained on the return voyage; but he increased the amount of chloral. He had again to cross the channel at the end of September, by the night boat from Calais to Dover, and thinking, with reason, that the sea would be rougher at that season than usual, he had a draught made up composed of chloral, 3 grams, (45 grains;) distilled water, 50 grams; gooseberry syrup, 60 grams; and French essence of peppermint, 2 drops. He took half the draught as the vessel left the harbor, and arrived at Dover without having suffered in the least from sea-sickness, whilst his companions were in the usual condition of prostrate misery. A very heavy sea was running. On his return from London on October 30, there was a high sea and much wind; he accordingly took the remaining portion of his draught, soon went to sleep, and only awoke on his arrival at Calais in the best possible condition. Dr. Giraldes remarks that he is, as a rule, affected by sea-sickness when he crosses the channel, and that his two trials of chloral have convinced him of its efficacy as a preventive of that most disagreeable malady. He adds that he never goes down into the cabin, but makes himself as comfortable as circumstances will allow on deck.—*Medical News.*

*Cinquefoil in Puerperal Peritonitis, and on the Treatment of Diphtheria.*—By WM. HAUSER, M. D., of Bartow, Jefferson County, Ga. In the United States Dispensary, edition for 1851, this plant is characterized thus: "*Potentilla, Reptans Cinquefoil,* a perennial, creeping, European herb, with leaves which are usually quinate, and have thus given origin to the ordinary name of the plant.—The root has a bitterish, styptic, slightly sweetish taste, and was formerly used in diarrhœa, and other complaints for which astringents are usually prescribed."

I shall say nothing of the properties here ascribed to this plant, but I will say that it is the best and most powerful *sudorific* I have ever found. And, like all of its class, it is, under certain circumstances, diuretic also. Dr. Edwin LeRoy Anthony, son of Dr. Milton Anthony, founder of the Medical College of Georgia, assured me, many years ago, that he had cured gonorrhœa with it.—But my purpose, in this short article, is to ask the attention of the medical profession to it in treatment of peritonitis of any kind, but specially *puerperal peritonitis.* In a large practice, of more than twenty years, I have never found anything, nor all other things combined, to equal this simple plant in the treatment of this exceedingly painful, dangerous and sometimes stubborn disease. I have never failed with it once, in all this time, to the best of my recollection. A recent case that gave much trouble and anxiety to two of my honored medical brethren, has brought it afresh to my mind; though I have not been in practice myself for eight years. My method with it is simply this: Make as strong a decoction of the plant, leaves, vines and roots, as possible, and give the patient (at any stage of the case) large draughts of the tea, as hot as she can drink it, every half hour, or oftener, till she be thrown into full perspiration. All pain and fever will soon be gone, and then you have the entire mastery of the case.

This plant is *American*, not European, and resembles the common strawberry, in leaves, vine, and mode of bunching. Its blossoms are yellow.

*Diphtheria.*—I shall not waste the time of the printers and readers of the *Journal* by writing a word on the characteristics of diphtheria, as I hope every reader has carefully studied Dr. George E. Trescot's scholarly and exhaustive article on the subject; I wish only to tell in the plainest style, and fewest possible words, how I have cured it, in every case I have treated. Here is the plan: ℞. Nicotiana, tabacum, opt., Piper rubrum, aa. ʒij.; Sodæ muiras, ℥ij. Pour boiling water on these articles, about two gills, or more. Mop the tonsils and neighboring parts frequently, severe as it will be, till relief is obtained. Be bold, and fear not; nay, "doubt not." If half a grain of solid carbolic acid be added to the above, all the better. Mop when the decoction is blood-warm.

*Proposed Standard for the Doctorate.*—At a late meeting of the San Francisco Medical Society, the following preamble and resolution, proposed by Dr. John F. Morse, were, after discussion, adopted, and commended to the consideration of the several medical associations throughout the State:

WHEREAS, There seems to be a prevailing conviction in our profession that a higher standard of medical education is required, for the good of its members and the security of the public; and whereas, the agitation of this subject, annually encouraged in the American Medical Association, though resulting in emphatic declarations of the necessity, have thus far signally failed to inspire any effective or practical effort at improvement; and whereas, anything which effects a more independent and careful examination of mature medical students must tend to stimulate teachers and pupils to the greatest degree, must make more glorious the office of teaching, more efficient the struggles of emulating medical colleges, must end in the securement of the best possible professors, the most thorough students, and in evolving the best system of medical education for enhancing the guarantees of graduation and the value of medical diplomas: therefore—

*Resolved*, That, in the opinion of this Medical Society, there should be a competent, independent State Board of Medical Examiners, whose duty it should be to carefully examine all persons who claim the proper qualifications, and who desire to obtain a diploma of regular medicine; and that to such applicants as pass this examination and receive the indorsement of the aforesaid Board of Examiners, there should issue a diploma from the highest possible State authorities, irrespective of any conditions except the thorough qualification of the applicant, as attested by the Board of Examiners.—*Pacific Medical Journal.*

*Apples in Paris.*—The *New York Medical Record* is authority for the statement that, according to an ancient French physician, the growing immunity from dyspepsia and hepatic derangements in Paris, is due to the increased consumption of apples, which, it is claimed, are excellently prophylactic and tonic, besides possessing value as a nourishing and easily digested article of food. The capacity of the Parisians is said to be 100,000,000 every winter.

J. B. M.

*Grindelia Robusta in Asthma.—By Q. C. Smith, M. D., Cloverdale, Cal.*—Several months since, at the suggestion of Dr. W. P. Gibbons, of Almeda, we procured a small package of the solid extract of grindelia robusta, with the intention of giving it a trial in the treatment of diseases of the respiratory organs. And as Dr. Gibbons desired us to report the results, we take this opportunity of doing so. We have used the remedy in one case only, and that was a case of spasmodic asthma. We could detect no organic lesion, either in the lungs or any other part of the patient's body.

Mr. M., the patient referred to, is thirty-seven years of age, tall, rather spare habit, with narrow chest; a stock rancher by occupation; has lived in California since he was eleven years of age; born in Missouri, and lived there prior to his emigration to this State. Has suffered from severe and frequent attacks of asthma since early childhood, for the relief of which he has taken many remedies from many physicians and non-professional persons, with little and only temporary and partial relief, never being entirely free from asthmatic symptoms. Much of the time he was unable to walk more than a few paces at a time without stopping to get breath. About five months since he commenced to take the extract grindelia in pills; taking, when the attacks came on, two or three grains three times a day, for two or three days, then taking two or three grains at bed-time only, for eight or ten days longer.

Under this mode of treatment, the attacks became lighter and more remote; and, during the interval between the attacks, he gained flesh and strength, and improved greatly in his general health.—*Pacific Medical and Surgical Journal.*

*Prolapsus Ani Treated by Injections of Ergotine.*—Von Langenbeck, of Berlin, announces that he has lately been treating prolapsus ani with astonishing success, by hypodermic injections of a solution of ergotine (five to fifteen parts to one hundred of distilled water.) He replaces the bowel, and inserting the point of the syringe about three centimetres in depth in the cellular tissue, throws in from one to two grains of ergotine. This should be repeated every three or four days, for three or four weeks, any hard fæcal masses in the bowel being first removed by simple injection.—*Medical Press.*

*Belladonna in Spasmodic Asthma.*—Dr. George G. Wood, in the *Philadelphia Medical Times,* of the 19th of September, gives the result of his experience with large doses of belladonna in the treatment of asthma. He says:

"I usually employ the tincture of the United States Pharmacopœia, in doses ranging from twenty to sixty drops. The strength of the tincture differs so much, as commonly kept in shops, that the size of the dose must be lost sight of, and the quantity given be regulated by the effect produced. It may be given during the paroxysm with great advantage, but it acts best when given before the attack commences. For example, if the patient has nocturnal attacks coming on after midnight, as is usual, give him a dose just before going to bed, and repeat it if necessary to produce sound sleep. He fails to awake at the usual time for the attack to commence, and sleeps on, awakening in the morning very much refreshed and strengthened. This treatment may be repeated night after night, until sufficient time has been had to remove the tendency of the disease to return, either by changing his location, or adopting other requisite treatment as the case may call for. I could relate several cases to prove the above statement, but will have to omit them for want of space.

"Sometimes, but not often, bellodonna produces dryness of the fauces, and delirium. These are indications which show that it should be discontinued, and hydrate of chloral should be employed in its stead. It may be used on the same principles as belladonna to produce sleep, and thus ward off attacks. For the past two years I have been treating spasmodic asthma on these principles, and with most satisfactory results."—*New York Medical Journal.*

*Solution of Morphia for Hypodermic Injection.*—Dissolve ten grains of hydrochlorate of morphia in two drachms of distilled water by the aid of heat, without any acid, spirit, or glycerin. Two minims of this solution, *i. e.* one-sixth of a grain, should be the commencing dose. It becomes solid at ordinary temperatures, and when wanted for use must be heated. The advantage is, that however long it is kept, the solution never spoils.—*Dr. H. Lawson, in Canada Lancet.*

*Treatment of Pertussis by Inhalation.*—Dr. J. Winthrop Spooner, in the *Boston Medical and Surgical Journal*, November 5th, 1874, details the results of his experience in eleven cases of whooping-cough treated by the plan recommended by Dr. John J. Caldwell, of Baltimore, in the number of that journal for April 20th, 1871; viz: ℞. Fluid-ext. belladonnæ, mv ad x; potass. bromidi, ℈i; ammon. bromidi, ℈ij; aquæ, ℥ij. Inhale one table-spoonful in an ordinary steam-atomizer. Dr. Spooner uses a tablespoonful of this mixture, and fills up the glass of the atomizer with water. When the disease is at all severe, he uses the atomizer twice daily until the urgency of the symptoms is relieved, and then continues it once daily until the cough has entirely disappeared. In some cases, he has somewhat varied the proportion of the ingredients, but has made no essential departure from the formula given: The effect of the method shows itself immediately; and, besides the distressing symptoms, the period ot the disease itself is much lessened in the majority of cases. In only one of the eleven cases was any other treatment than that by inhalation used; and the apparent failure in this casc seemed to be due to the difficulty in administering the remedy thoroughly, on account of the age of the child—only two years old.—*British Medical Journal.*

*Extraction of Foreign Bodies from the Eye.*—A correspondent of the *Lancet* writes as follows:

"Sir—In consequence of the difficulty I experienced in removing from a patient a portion of steel deeply imbedded in the cornea, which did not yield to spud or needle, some other means for its removal became necessary. Dry, white soft silk waste suggested itself to me, and was wound round a thin piece of wood, so as completely to envelop its end. This soft application was then brushed once backwards and forwards horizontally over the part of the cornea where the foreign body remained fixed. To my astonishment, it was at once entangled by the delicate but strong meshes of the silk and was withdrawn with the greatest ease, caught by the same.—Seeing no books (which I have consulted) mention this application, and feeling sure it is one of great value in removing foreign bodies fixed or floating in the conjunctiva or cornea, it seems to me a ismple but valuable invention."—*Medical Times.*

*Studies and Experiments on the Prevention of Contagion in Disease.*—By Dr. JUSTINIAN V. FROSCHAUER (*Wiener Medicinisch-Chirurgische Rundschau.*)—Translated from the German by J. H. Carstens, M. D.)—The author obtained ten healthy lambs from a region perfectly untainted by disease. These lambs he vaccinated with sheep-pox lymph. Four of these ten lambs (who were kept in an ordinary stable) died in from sixteen to twenty-two days, with all the appearance of pox. The other six he kept in an atmosphere containing sulphureted hydrogen ($\frac{1}{4000}$). Excepting one lamb, which was slightly sick, all remained perfectly healthy, even in the places where the virus was introduced.

This immunity from pox in the lambs, the author thinks, is due to the sulphureted hydrogen. Like vaccine, other less injurious substances can protect against severer ones.

The author also subjected rabbits to an atmosphere containing sulph. hydrogen and then injected subcutaneously a dose (sufficient to kill) of cyanide of potassium; the rabbits lived, and the author concludes that the preparatory treatment caused the immunity from the deadly dose of potassium cyanide.

As it is impossible to follow and destroy contagion, the author lays special stress on the necessity of producing an immunity from disease (cholera, etc.). This immunity can be produced, as he thinks, by a relatively small quantity of sulphureted hydrogen.

The author requests that experiments be made on animals suffering from rinderpest, etc.—*Detroit Review.*

*Death after Delivery, without any Lesions to account for it.*—The President recited the history of the case of Lizzie Stern, who died recently on board the steamer Bellevue, after being delivered of a child. She was taken to the steamer by the ambulance, to be conveyed to the Charity Hospital, Blackwell's Island, and when on board of the steamer was confined. For half an hour after delivery she was apparently well, but then began to sink, and died in two hours. There was no hemorrhage.

*Autopsy.*—All of the organs were in a state of health. The blood was fluid, and the heart did not contain a clot.

The point of interest in the case was the sudden death, without any obvious lesions to account for it.—*New York Medical Journal.*

*Passage of a Scissors through the Abdominal Walls, after being Swallowed.*—Dr. H. B. Sands (*New York Med. Journal*, December, 1874) details a case in which, while affected by a suicidal mania, a patient swallowed a scissors. Patient told her attendants that she had done so; but being addicted to lying, she was not believed. Some time after this she complained of pain in the right side, above the umbilicus, and to the right of the median line. A tumor appeared, which was poulticed; an ulcer formed, and it was then found that the points of the scissors were protruding. From this opening intestinal and biliary matters came away, which showed a communication with the pyloric extremity of the stomach, if not the small intestine. It was found that the scissors as presenting, could not be removed, but by unfastening the rivet, one blade and then the other was successfully removed. The blades were five inches long and three-fourths of an inch wide. Twelve hours after the exit of the last blade there was no trace of biliary or intestinal matters, and in two days the wound had completely healed up, and very shortly she was entirely well.—*Detroit Review.*

This case is singular, and contains a moral. If the patient had not been "addicted to lying" the scissors might have been removed at once, and she would have been saved much pain and danger. Patients, don't lie; at any rate, don't lie to your doctor.

H. B. L.

*Life in a Six Months' Fœtus.*—Dr. W. L. Atlee (*Medical Times*, February, 1875) reports the following case observed by himself, in 1845: At closest calculation, the period of gestation did not exceed six months. The very small child being flaccid and apparently lifeless, was rolled in a cloth and laid one side. The placenta being removed, and mother made comfortable, he was asked the sex of the child. On unwrapping it, he observed a slight gasp. It was now laid in a bed of cotton wadding, and its grandmother took charge of it, feeding it by dropping milk into its mouth from the point of her finger. After being kept and fed in this way for two weeks, it was first washed and dressed. At that time it weighed two and one-fourth pounds. It lived, and is now a beautiful and vigorous lady.—*Detroit Review.*

*Chloride of Zinc.*—Dr. J. E. Nichols, in the *Chicago Medical Journal*, for May, gives his experience in the use of chloride of zinc in cancer, indolent ulcers and tumors. He directs the zinc to be rubbed up with powdered b'ood-root, using enough of the blood-root to take up the moisture caused by the deliquescence of the zinc, so as to form a plastic dough, firm enough not to spread beyond the limits desired. In using, protect the adjacent parts with adhesive plaster. If the first eschar is not deep enough, use again, or substitute Dr. Marsden's arsenious mucilage: ℞. Arsenious acid, ʒij; mucilage, gum acaciæ, ʒi; m. ft. a stiff paste. Apply this to not more than one square inch of surface, and cover with dry lint. Either of these pastes may be left from six to forty-eight hours, according to the impression desired. Until the eschar comes off poultices are the best dressing; after, use simple cerate. Dr. N. has used this treatment with perfect success in chronic anthritis arising from injury, a cure being obtained by two applications. He has also used it in several cases of *rodent ulcer*, or lupus—also known as "*noli me tangere*"—of several years duration; also in cases of cancer, instead of the knife, with apparent success. H. B. L.

*Is Puerperal Fever Contagious?*—A writer in *London Lancet* cites the following illustration on this point, and at the same time gives a practical illustration of an admirable method of limiting it to the original patients. "Three years ago an outbreak of puerperal fever took place in a provincial town of considerable size. On investigation it was found that all the cases had occurred in the practice of one surgeon and one midwife. The members of the profession, located in the town, met and requested the gentleman in question to refrain from practicing midwifery for one month, and at the same time agreed to attend his cases for him. The midwife was likewise requested not to attend cases for a similar period. This was agreed to, and no new cases occurred after that date."

This seems almost conclusive proof that puerperal fever is contagious. All physicians should so regard it, and take the necessary steps to prevent the spread of this terrible disease.—*Detroit Review.* H. B. L.

*Successful Cæsarean Section in a Case of Uterine Fibroids.*—Dr. Cazin has had occasion to practice the Cæsarean operation in a case of uterine fibroids, and has had the good fortune to save the mother and child. He operated on a woman, aged thirty-nine, in whom, towards the sixth month of pregnancy, fibroid tumors were recognized in the posterior and inferior wall of the uterus. Labor set in the seventh month; after four days of pains the membranes ruptured, and a hand escaped, the child being still alive; but as it could not be extracted, either by forceps or by version, recourse was had to the Cæsarean operation. The most minute precautions were taken; there were hemorrhage and syncope, inertia of the uterus, distention of the belly to such a degree that it became necessary to puncture the bowel to give exit to the gas; there was vesical paralysis, and an abscess formed between the uterus and the abdominal wall. In spite of these complications the patient got well, and the child, baptized Cæsar, throve. It has since been ascertained that the fibroids are diminishing in size.—*Gaz. Med. de Paris.*

*Compound Tincture of Opium.*—(Diarrhœa Mixture. Formula by Dr. E. R. Squib.)—Take of, tincture of opium, 1 fluid ℥; tincture of capsicum, 1 fluid ℥; spirit of camphor, 1 fluid ʒ; purified chloroform, 3 fluid ʒ; stronger alcohol, sufficient quantity to make the measurement 5 fluid ounces. Each fluid drachm contains about one hundred drops, consisting of twelve drops each of the first three ingredients, and four and a half minims or eighteen drops of chloroform. Dose—One teaspoonful in water for adults.—*Druggists' Circular.*

*I. E. S.* (*Chicopese Mass.*)—Pharaoh's Serpents' Eggs are made of sulpho-cyanide of mercury mixed with mucilage to render it more cohesive. The salt is to be had of manufacturing chemists. The process to prepare it was given in *The Druggists' Circular* for May, 1874, page 97.—*Druygists Circular.*

*Tincture of Polk Root.*—(See proceedings of Am. Pharm. Association, 1875.) Take of poke root, in moderately fine powder, 6 troy ozs.; cardamoms, in fine powder, 120 grains.; diluted alcohol, 2 pints. Macerate fourteen days, and filter through paper.—*Druggists' Circular.*

*Bland on the Treatment of Scarlatina by Hyposulphites and Carbolic Acid.*—Dr. Bland says (*Transactions of the Medical Society of the State of Pennsylvunia*): In the treatment of scarlatina, anginosa, I must again renew my indorsement of the internal administration of the hyposulphites and carbolic acid. The results observed in this disease with the above remedies are unprecedented, and give every evidence to warrant their continued use.

I recently treated seven cases in one family, four of whom had severe throat-ulcerations. The topical application of carbolic acid and glycerin to the inside of the throat, the hyposulphite of soda internally, with good, nutritious diet, and febrifuge mixture as required, was the treatment employed, and in eight days the patients were all well. A number of my colleagues have tried the foregoing remedies and give them unqualified indorsement.—*St. Louis Medical Journal.*

*Mustard Foot-bath in Urticaria.*—A writer in the *Tribune Medicale,* March 7, recommends the use of the mustard foot-bath in cases of urticaria. In his own case, after trying innumerable remedies, he was about to abandon all hope of relief, when, one day, while suffering from a peculiarly aggravated attack of his old enemy, complicated by an excruciating headache, and hoping to relieve the latter, he plunged his feet into a mustard-bath. The relief was instantaneous, and it seemed as though the skin-disease had disappeared by a wave of the hand.

Five other cases were subsequently treated by the writer, with similar relief. The treatment is, of course, understood to be only palliative, and has no influence in preventing recurrence of the disorder. X.

*A Simple Method of Removing the Teeth of Children.*—The operation consists in simply slipping a rubber ring over the tooth and forcing it gently under the edge of the gum. The patient is then dismissed and told not to remove the appendage, which in a few days loosens the tooth and causes it to fall out. Grown children who shrink from the shock and pain of the dental nippers, may also have their teeth removed by means of the rubber, which is a mild form of treatment.—*Druggists' Circular.*

*Ablation of the Breast by the Elastic Ligature.*—M. Périer had under his care recently, at the Salpêtrière, an old woman suffering from a tumor of the breast (diagnosed cysto-sarcoma.)

This tumor, the size of the fist, mobile and detached by its own weight from the surrounding tissues, was removed by the elastic ligature. The base of the tumor was surrounded by the ligature, the ends of which were then tied under long pins previously introduced. The tumor preserved a red color for a few days, then became blue, and finally fell off. Unfortunately, the patient died two months subsequent to the operation, of erysipelas of the face.—The advantages of the operation are the avoidance of loss of blood, more rapid cicatrization than with the galvano-cautery, and the being able to do without anæsthetics.—*Bull. Gen. de Therap.*

*Apomorphine.*—This remedy, which in composition differs from morphine only in having one equivalent less of water, possesses properties totally different from the latter body. It exercises an elective and almost exclusive action on the nervous centres which control vomiting. Employed hypodermically, which is the best way of giving it, it produces vomiting in from six to ten minutes.—There is no subsequent sickness or irritating effect on the digestive tract. The dose for adults is 7 to 8 milligrammes, for children 1 to 2 milligrammes. M. Moeller recommends that the first injection should contain 5 milligrammes, to be repeated if vomiting does not ensue. M. Jurasz recommends apomorphine as an expectorant, and administers it in doses of from 1 to 3 milligrammes every two hours.—*Lyon Medicale.* H. F.

*Aromatic Syrup of Senna.*—(See Proceedings of Am. Pharm. Association, 1857.) Take of Alexandria senna, 4 troy ounces; jalap, 1½ troy ounces; rhubarb, ½ troy ounce; cinnamon, 60 grains; clove, 60 grains; nutmeg, 30 grains; oil of lemon, 20 minims; sugar, 24 troy ounces; diluted alcohol, a sufficient quantity. Reduce the root, leaves, and spices to a moderately fine powder, and treat with the diluted alcohol by percolation, to exhaustion; when about one quart has passed, evaporate by means of a water-bath to eighteen fluid ounces, and filter if necessary; then add the sugar, and dissolve by water-bath; when cold, add the oil of lemon. It should measure thirty-two fluid ounces.—*Druggists' Circular.*

*Aspirator in Retention of Urine.*—The usefulness of the aspirator in relieving retention of urine, when, for any reason, the catheter cannot be employed, has been demonstrated by an additional number of published cases, and in many more that have not been deemed worthy of recording, so well established has the operation become. M. Fochier has recently read quite an elaborate paper on the subject, urging its entire freedom from danger, even when, by accident, the peritonæum is punctured. One objection that has been urged against it is that a small amount of urine is liable to remain at the bottom of the bladder, and, there undergoing decomposition, to set up a cystitis. Dr. James A. Hall, of Elmira, N. Y., has pointed out that he has been able to avoid this, by laying the patient on his side, and emptying the bladder completely.—*New York Med. Journal.*

*Standard Weights and Measures.*—The American Meteorological Society, at its annual meeting, held at the Cooper Institute in this city on the 28th of last month, adopted the following resolutions:

*Resolved*, That this Society highly approves of the plan which has been set on foot among the members of scientific professions [illegible] the United States to secure a general consent to the adoption of the metric measures and weights in their professional practice after July 4, 1876.

*Resolved*, That the members of this Society will as far as they may be able, employ their personal influence in aid of the plan above mentioned.

*Resolved*, That a committee be appointed to recommend the erection in the various State capitals of a standard yard and metre, to be provided by the Bureau of Weights and Measures of the United States.

*Cure for Warts.*—Lisfranc immerses the parts on which the warts are developed in a strong solution of black soap. This causes a slight cauterization of the surface of the wart. The loosened tissue is to be removed and the application repeated every day till the cure is complete. Oil of vitrol should never be used for this purpose; it is very irritating and inflames the warts instead of curing them.—*Trib. Med.* C. R. C.

*Dislocation of Humerus after Five Months.*—Dr. Thomas Gregg communicated, to the south of Ireland branch of the British Association, (February 10th,) the case of a woman, admitted into the county hospital on January 28th, the injury having occurred on September 5th previously, from an accident which was caused by a threshing-machine, in which her arm was caught. The nature of the injury was overlooked. On presenting herself at the hospital, the usual appearance of dislocation under the clavicle, the head of the bone being fixed in this position, and adherent to the neck of the scapula. On February 3d, the reduction was effected under chloroform, with pulleys. The usual apparatus employed in the hospital for old-standing luxations of the shoulder joint, is a gutta-percha shield, which fits from spine to sternum, with an aperture for the arm, carefully moulded and bandaged to the body. This protecting shield affords a means of steadying the scapula, and also a fixing point for the straps, and preventing injury to the ribs.—The President cited several instances of old-standing luxation, which, he said, could not have been reduced but for the use of this gutta-percha shield.—*British Med. Journal.*

*Damiana.*—This Mexican plant, the technical name of which we are unable to present to our readers, is highly extolled by Dr. J. J. Caldwell, of Maryland, as a powerful aphrodisiac and general stimulant to all the urino-genital organs. The Doctor gives several cases in which its effects were very marked, and concludes his paper in these words: "I would recommend the tincture and fluid extract of damiana. I prefer the fluid extract, as it is less bulky, more positive in its effects and more reliable and uniform, as proven by cases now under my care. Indeed, this remedy seems to have a specific effect upon all of the organs of the pelvis, giving increased tone and activity to all of the secretions in that vicinity."—*Virginia Medical Monthly.* H. B. L.

*Chloral and Morphia in Tetanus.*—M. Bourdy, of Mans, has reported a case of tetanus successfully treated by chloral and morphine subcutaneously. The daily dose of chloral was 120 grains, and during the treatment the patient took seven ounces of chloral and 27 grains of morphine.—*New York Med. Journal.*

*Hypodermic Medication.*—The Standing Committee of the New Jersey State Medical Society, at the last annual meeting, reported the following conclusions as to this mode of medication:

1. It is very generally in the hands of the medical men of the State, and by many of them very extensively employed.
2. It is a safe remedial procedure.
3. It is chiefly valuable in subduing pain and suffering.
4. Morphia, in solution, is the drug most extensively employed, and, practically, the only one on which general reliance is placed.

The above *resumé* is from the reports of the district societies throughout the State, which were unusually interesting and full. Let those timid souls, if such there be, and we take it yet there are, who hesitate to avail themselves of this *immensely* beneficial method of relieving pain, captious critics to the contrary, notwithstanding, cast aside their fears, and advance themselves abreast the age. J. B. M.

*Morphia with Atropia for Subcutaneous Administration.*—We highly recommend this combination. We have used it satisfactorily for years. Its object is three-fold. We regard the effect of the morphia enhanced thereby. We feel confident that any overaction of either, if given alone, will be held in check by the antidotal properties of the other. Besides, we think atropia exercises a marked control, though not specific, over the gastric irritability so often resulting from morphia alone administered. Dr. Finney recommends atropia gr. 1-30 to morphia 1-4. We deem this dose of the former unnecessarily large. Our usual quantity is gr. 1-60, which, in the majority of cases, will be found productive of atropism, to the extent of faucial dryness and moderate pupillary dilatation. J. B. M.

*Cough and Sweating in Phthisis.*—Dr. Little, of Dublin, recommends the following combination for the relief of the distressing cough of phthisis, and for diminishing the sweating: Acetate of morphia, 2 grains; liquor of atropia, 6 minims; dilute hydrocyanic acid, 35 minims; syrup of Virginia prune to an ounce and a-half. A measured drachm to be taken, unmixed with water, on going to bed, and once again during the night if necessary.—*Dublin Journal of Medical Science, January,* 1875.

